# 4D visualization of Alzheimer's Disease Progression for Clinical Assessment

**DOI:** 10.1002/alz70856_102920

**Published:** 2025-12-26

**Authors:** Ying Weng, Ke CHEN, Poosanu Thanapornsangsuth, Yiming Zhang

**Affiliations:** ^1^ University of Nottingham Ningbo China, Ningbo, Zhejiang, China; ^2^ Division of Neurology, Department of Medicine, Faculty of Medicine, Chulalongkorn University, Bangkok, Thailand

## Abstract

**Background:**

Alzheimer's disease (AD) is a chronic neurodegenerative disorder that is characterized by the presence of abnormal proteins in the brain. With its progression, the disease affects all the major brain areas, which causes the problems in memory, thinking, judgment ability, speech, logic, personality, and movement. Cognitive function typically decreases under such conditions. As a major cause of dementia, AD is a concern for millions of people in the world. This also makes it a huge economic problem for families and healthcare systems. Therefore, accurately monitoring AD progression is crucial for clinical assessment with timely drug interventions to develop personalized treatment plans and deliver effective treatment.

**Method:**

In this study, we present a computer‐assisted system that uses novel 4D monitoring technologies for intelligent AD progression. In this system, we combine 3D magnetic resonance imaging (MRI) spatial data with an addition of 1D temporal data into spatio‐temporal 4D dynamic visualization for allowing the analysis of brain changes over time. We develop the 4D visualization system with the 3D Slicer software, which is a user‐friendly platform for clinical assessment, and enables clinicians to visualize AD progression more accurately.

**Result:**

We perform various experiments to assess the efficiency of the proposed 4D visualization computer‐assisted system for intelligent monitoring AD progression. We utilize ADNI dataset, which contains brain imaging data, biofluidic biomarkers, cognitive assessments, genetic information, and demographic data. Hereby, the MRI image data from ADNI are deployed to show the progression of AD. The system allows interactive 4D dynamic visualizations for up to 160 months of 3D MRI brain images for AD patients in the ADNI dataset. The experimental results are promising, which indicates that our proposed technique is capable of helping clinicians identify AD progression by detecting alterations in brain structure over time.

**Conclusion:**

The study presents an intelligent computer‐assisted system based on 4D visualization for AD progression. The system is helpful to advise clinical assessment with critical information which is beneficial for diagnosis and drug treatment plan based on monitoring of AD progression.

